# In Vitro Characterization of Gut Microbiota-Derived Commensal Strains: Selection of *Parabacteroides distasonis* Strains Alleviating TNBS-Induced Colitis in Mice

**DOI:** 10.3390/cells9092104

**Published:** 2020-09-16

**Authors:** Bernardo Cuffaro, Aka L. W. Assohoun, Denise Boutillier, Lenka Súkeníková, Jérémy Desramaut, Samira Boudebbouze, Sophie Salomé-Desnoulez, Jiří Hrdý, Anne-Judith Waligora-Dupriet, Emmanuelle Maguin, Corinne Grangette

**Affiliations:** 1 Université de Lille, CNRS, Inserm, CHU Lille, Institut Pasteur de Lille, U1019-UMR 9017-CIIL-Centre d’Infection et d’Immunité de Lille, 59000 Lille, France; bernardo.cuffaro@gmail.com (B.C.); Denise.boutillier@pasteur-lille.fr (D.B.); jeremy.desramaut@pasteur-lille.fr (J.D.); 2 Institut Micalis, MIHA Team, Université Paris-Saclay, INRAE, AgroParisTech, 78350 Jouy-en-Josas, France; landrywil05@yahoo.fr (A.L.W.A.); samira.boudebbouze@inrae.fr (S.B.); 3 Laboratoire de Biotechnologie et Microbiologie des Aliments, UFR en Sciences et Technologies des Aliments, Université Nangui Abrogoua, Abidjan 00225, Côte d’Ivoire; 4 Institute of Immunology and Microbiology, First Faculty of Medicine, Charles University and General University Hospital, 121 08 Prague, Czech Republic; lenka@sukenikova.com (L.S.); jiri.hrdy@lf1.cuni.cz (J.H.); 5 Université de Lille, CNRS, Inserm, CHU Lille, Institut Pasteur de Lille, US 41-UMS 2014-PLBS, 59000 Lille, France; Sophie.SALOME-DESNOULEZ@ibl.cnrs.fr; 6 UMR-S1139 INSERM, Faculté de Pharmacie de Paris, Université de Paris, 75006 Paris, France; anne-judith.waligora@parisdescartes.fr

**Keywords:** microbiota, probiotics, holobiont, live biotherapeutic products (LBP), IBD, immune response, colitis, functional screening

## Abstract

Alterations in the gut microbiota composition and diversity seem to play a role in the development of chronic diseases, including inflammatory bowel disease (IBD), leading to gut barrier disruption and induction of proinflammatory immune responses. This opens the door for the use of novel health-promoting bacteria. We selected five *Parabacteroides distasonis* strains isolated from human adult and neonates gut microbiota. We evaluated in vitro their immunomodulation capacities and their ability to reinforce the gut barrier and characterized in vivo their protective effects in an acute murine model of colitis. The in vitro beneficial activities were highly strain dependent: two strains exhibited a potent anti-inflammatory potential and restored the gut barrier while a third strain reinstated the epithelial barrier. While their survival to in vitro gastric conditions was variable, the levels of *P. distasonis* DNA were higher in the stools of bacteria-treated animals. The strains that were positively scored in vitro displayed a strong ability to rescue mice from colitis. We further showed that two strains primed dendritic cells to induce regulatory T lymphocytes from naïve CD4^+^ T cells. This study provides better insights on the functionality of commensal bacteria and crucial clues to design live biotherapeutics able to target inflammatory chronic diseases such as IBD.

## 1. Introduction

The mammalian gut harbors a complex and dynamic community of microorganisms, collectively called the gut microbiota, including archaea, fungi, viruses, protists, helminths and mainly bacteria [1,2]. Among bacteria, Bacteroidetes and Firmicutes are the most abundant phyla together with less abundant Proteobacteria, Actinobacteria, Fusobacteria and Verrucomicrobia. Despite a high inter-individual variability, metagenomic analysis highlighted a core functional gut microbiome including approximately 60 bacterial species shared by healthy subjects [2,3,4]. This microbial community lives in a symbiotic relationship with the host displaying important metabolic, immunologic and gut protective functions [5,6]. It notably exerts a pivotal role in educating and orchestrating both the innate and adaptive immune response at the intestinal level. Indeed the microbiota plays a key role in maintaining the intestinal epithelial barrier function, the immune homeostasis and the protection against pathogen colonization [7].

Alterations in microbiota composition together with reduced bacterial diversity, known as dysbiosis, are frequently observed in patients suffering from a variety of chronic disorders including inflammatory bowel disease (IBD) [8,9,10], obesity [11,12,13], type 1 and 2 diabetes [14,15]. However, it remains difficult to establish a definitive causal role of the gut microbiota to such diseases. The role of bacteria in IBD has been strongly supported in animal models in which no colitis occurred after eradication of the colonic microbiota by antibiotics or the use of germ-free animals [16,17]. Moreover, microbiota transplantation from IBD patients or colitic animals to axenic recipient mice led to gut inflammation arguing that dysbiotic microbiota play an important role in the pathogenesis of inflammatory disease [18,19]. Despite fast gathering of omics data documenting the change of the intestinal microbiota in IBD, consensus about specific disease-relevant taxa remains weak, even if dysbiosis has been linked with the expansion of some pathobionts notably belonging to *Enterobacteriaceae* in about 30% of patients suffering from Crohn’s disease (CD) [20,21] and some bacterial [22] and fungal signatures [23]. Various studies identified decreased abundant species in IBD compared to healthy control, notably *Blautia faecis*, *Roseburia inulinivorans*, *Ruminococcus torques* and *Clostridium lavalense* [24]. CD is mainly characterized by the alteration in Firmicutes abundance, especially a reduction in *Clostridium* cluster XIVa and IV, with *Faecalibacterium prausnitzii* as the dominant species [8,25,26]. However, changes in the gut microbiota composition in IBD remain inconsistent and present many variations among the surveying studies. Therefore, prospective studies must be undertaken, notably with individual bacterial species, in order to identify the exact role of selected bacterial strains. *Parabacteroides* belongs to the human core intestinal microbiota [15,27] and to the 26 bacterial genera significantly enriched in the microbiome of healthy controls compared with patients affected by CD [22]. The therapeutic strategies for patients with IBD, mainly based on the use of anti-inflammatory and/or immunosuppressive drugs are not curative. In addition, about 25–30% of patients fail to respond to treatment and 20% of the patients will discontinue therapy due to side-effects [28,29]. Interestingly, patients who underwent biologic therapies, such as anti-TNF [30] or Adalimumab [31] showed a decreased gut microbiota dysbiosis, providing a rationale for the development of microbiota-targeting therapies.

Therefore, targeting the microbiota dysbiosis is becoming a forefront of biomedical research. Fecal microbial transplantation (FMT) has emerged as a putative microbiome-targeting therapy in IBD [32], however, determining the best donor and standardization of the protocol are still a challenge as well as the long-term effects resulting from the uncharacterized nature of FMT [33]. In this context, the use of probiotics defined as “live microorganisms that, when administered in adequate amounts, confer a health benefit on the host” [34] has received a lot of attention in the last decade because of the “natural” and safety aspects of such a treatment [35]. Traditional probiotics are indeed based on their classification as QPS (qualified presumption of safety) by the European Food Safety Authority (EFSA) or GRAS (Generally Recognized as Safe) by the United States Food and Drug Administration (FDA). They included a limited number of genera, mainly belonging to *Lactobacillus* spp. and *Bifidobacterium* spp. with a long history of use with proved biological safety. Many beneficial effects have been attributed to such microorganisms using various in vitro and animal experimental models. We and others have reported beneficial impact of traditional probiotics administration in mice, highlighting their capacity to promote immune regulatory responses [36,37] and epithelial barrier strengthening [38,39]. Systematic review of randomized controlled trials reported some efficacy of probiotics in induction or maintenance of remission in ulcerative colitis and pouchitis, however data are insufficient to recommend probiotics for use in CD [40,41].

Based on this low effectiveness and considering the increased knowledge about the gut microbiota, identification and characterization of novel and disease-specific health-promoting bacteria, commonly called next generation probiotics (NGP), start to emerge as new preventive and therapeutic tools. Since they did not have the same long history of safe use as traditional probiotics, they are now better defined as live biotherapeutic products (LBPs) or Pharmabiotics [42]. Such LBPs selected from microbiota microorganisms were showed to exhibit health-promoting properties in the management of chronic diseases such as obesity and metabolic disorders [42,43,44,45,46,47,48]. Regarding IBD and IBS, the beneficial effects of *F. prausnitzii* as a NGP have been successfully shown in several experimental models [49] and is currently under clinical investigation.

These results provided a rationale for the use of bacteria isolated from the gut microbiota as a source of LBP for the prevention or treatment of chronic diseases associated with microbiota dysbiosis. To achieve this goal, isolation and screening of new strains by stringent functional validation have to be performed, including the use of in vitro models followed by in vivo experiments and safety evaluation before performing validation in human clinical trials [50]. We performed an extensive prospective in vitro screening study on thirty strains of different species that originated from the human gut and highlighted the health-promoting potential of *P. distasonis* species (manuscript in preparation). We notably highlighted their anti-inflammatory profiles and their abilities to restore the epithelial barrier. Based on these results, we compared in the present study, the functional properties and possible strain phenotypic variability of a set of five different *P. distasonis* strains and evaluated in vivo their functional activities. This was achieved by combining in vitro approaches to evaluate their abilities to survive to the gastric conditions, to strengthen the epithelial barrier and their anti-inflammatory capacities. We then evaluated their abilities to prevent intestinal inflammation in a murine model of colitis and demonstrated high potential of two strains, supporting their future use as LBPs in the management of IBD. Among these strains, two were isolated from adult feces while three were isolated from newborn feces and all were assigned to the *P. distasonis* species through 16S V3–V4 region sequencing (data not shown).

## 2. Materials and Methods

### 2.1. Bacterial Strains and Growth Conditions

Bacteria evaluated in the present study are listed in Table 1. *P. distasonis* strains were cultured at 37 °C in the brain–heart infusion medium (BHIS) supplemented with yeast extract (0.5%, Difco), hemin (0.5 mg/mL; Sigma-Aldrich, Saint Louis, MO, USA), maltose (0.5 mg/mL; Sigma-Aldrich), cellobiose (0.5 mg/mL; Sigma-Aldrich), cysteine (0.5 mg/mL; Sigma-Aldrich) and K1 vitamin (0.098 mg/L; Sigma-Aldrich) in an anaerobic chamber (Jacomex, Dagneux, France) supplied with BIO300 (Air Liquide, Paris, France). After centrifugation (6000× *g* rpm, 15 min at 4 °C), culture pellets were washed with phosphate buffered saline (PBS; pH 7.2) maintained in anaerobiosis and bacteria were concentrated by centrifugation at 6000× *g* rpm for 15 min at 4 °C.

For in vitro experiments, cell pellets were suspended at 10^9^ CFU/mL in anaerobic PBS containing 25% glycerol and suspensions were frozen in liquid nitrogen before storage at −80 °C. For in vivo experiments, dry pellets (without PBS) were frozen in liquid nitrogen and stored at −80 °C.

Two strains were used as control strains for the peripheral blood mononuclear cells (PBMC) stimulation test. *Lactobacillus acidophilus* NCFM kindly provided by DuPont™ Danisco (Madison, WI, USA) and *Bifidobacterium animalis* spp. *lactis* BB12 provided by Gabriel Vinderola (INLAIN, UNL-CONICET, Santa-Fe, Argentina) were included respectively as pro-Th1 [51] or anti-inflammatory reference strains [52]. NCFM was grown under limited aeration at 37 °C in in De Man, Rogosa and Sharpe broth (MRS, Difco, Detroit, MI, USA) and BB12 was grown anaerobically (GENbag anaer, Biomérieux, Marcy l’Etoile, France), in MRS supplemented with 0.05% l-cysteine-hydrochloride (Sigma, Saint Louis, MO, USA). After overnight culture, bacteria were washed twice and resuspended in PBS buffer (pH 7.2) at 10^9^ CFU/mL for in vitro studies.

### 2.2. Resistance to Gastric Conditions

The survival kinetic of the strains was measured during 2 h of incubation in simulated gastric juice (SGF), as described in a consensus paper reported by an international network working in the field of digestion [53]. Briefly, SGF is composed of KCL 6.9 mM, HCl 15.6 mM, KH_2_PO_4_ 0.9 mM, NaHCO_3_ 25 mM, NaCl 47.2 mM, MgCl_2_ 0.1 mM and (NH4)_2_CO_3_ 0.5 mM and adjusted to pH 3 using HCl 1 M. CaCl_2_ was added to achieve a final concentration of 0.075 mM and porcine pepsin (Sigma) at 2.000 U/mL in the final digestion mixture. Bacteria were grown anaerobically at 37 °C in the BHIS medium. Cells were harvested by centrifugation (6000× *g* rpm for 10 min), washed twice with PBS (pH 7.2) and suspended in 0.2 mL of PBS. The bacterial suspensions were standardized at 10^9^ CFU/mL. 950 μL of SGF (with pepsin and at pH 3) was inoculated with 50 μL of the bacterial suspension and incubated at 37 °C during 2 h with sampling at time zero and then every 30 min. Bacterial viability was measured by numeration of serial dilution plated on BHIS-Agar after 48 h incubation in anaerobiosis. The death rate is calculated by dividing the number of CFU/mL at a given time point by the CFU/mL measured at time zero.

### 2.3. The In Vitro Epithelial Barrier Model

The epithelial barrier model was performed using the human colon epithelial cell line Caco-2 clone TC7 [54]. Cells were grown in Dulbecco’s modified eagle medium (DMEM, Life technologies, Grand Island, NE, USA) supplemented with 5% heat-inactivated (FCS), 1% non-essential amino acids, 2 mM glutamine, 100 U/mL penicillin and 100 μg/mL at 37 °C under 10% CO_2_. For the permeability test, cells were expanded on Transwell^®^ insert filter (polycarbonate membrane of 0.4 μm pore size, 12 mm diameters; Costar, Corning Life Science, Kennebunk, USA) starting at a density of 10^5^ cells per cm^2^, as previously described [38]. When optimal trans-epithelial electrical resistance was reached (TEER ≥ 1800 Ω/cm^2^ measured using a millicell Electrical Resistance System; Millipore, Billerica, MA, USA), fresh medium was added and cells were subsequently treated (or not) in the apical compartment, with the selected bacteria (bacteria-to-cell ratio of 10:1). Thirty minutes after, cells were sensitized with hydrogen peroxide (H_2_O_2_, 100 μM final concentration), in both apical and basal compartment. TEER was measured just before H_2_O_2_ addition (T0) and every 30 min until 180 min, and results were expressed in % TEER compared to T0. Three different experiments were performed including duplicates of each condition.

### 2.4. In Vitro Immunomodulation Assay

After approval of our experimental protocol by our institution committees (Institut Pasteur de Lille, agreement N° DC 2013-2022) in accordance with relevant guidelines and regulations, blood samples were collected from five healthy donors, after signed agreement. Peripheral blood mononuclear cells (PBMCs) were isolated after Ficoll gradient centrifugation (GE Healthcare Bio-Sciences, Uppsala, Sweden), as described before [51]. Cells were washed and adjusted to 2 × 10^6^ cells/mL in RPMI 1640 (Gibco, Life Technologies, Ghent, Belgium) supplemented with 150 μg/mL gentamicin, 2 mM glutamine and 10% heat-inactivated FCS (Gibco, Life Technologies, Grand Island, NE, USA). PBMCs were stimulated with PBS or bacteria (ratio cells/bacteria of 1:10) four 24 h at 37 °C under 5% CO_2_. Supernatants were collected, clarified by centrifugation and stored at −20 °C. Cytokine measurements were performed using R&D Duoset ELISA kits (R&D, Minneapolis, MN, USA).

### 2.5. Murine Model of 2,4,6-Trinitrobenzenesulfonic Acid (TNBS)-Induced Colitis

Animal experiments were performed in compliance with European guidelines of laboratory animal care (number 86/609/CEE), French legislation (Government Act 87–848) and approved by local Animal Ethics Committees (Nord-Pas-de-Calais CEEA N°75, Lille, France) and the Ministère de l’Education Nationale, de l’Enseignement Supérieur et de la Recherche, France (accredited No. 201608251651940). BALB/C ByJ mice (female, 7–8 weeks old) were obtained from Charles River (L’Arbresle, France) and were housed in specific pathogen-free condition at the animal facility of the Institut Pasteur de Lille (accredited No. C59-350009) under a temperature-controlled (20 ± 2 °C) environment and a 12 h light/dark cycle. Mice (*n* = 9 mice per group) were given ad libitum access to regular mouse chow (Safe, Augy, France) and water. After one week acclimation, the animals were treated daily by the selected bacteria (intragastric administration of 1 × 10^9^ CFU/mice in 200 μL gavage buffer composed of 200 mM NaHCO_3_/1% glucose) or gavage buffer alone (for control healthy mice and TNBS control mice, 5 days before until 1 day after colitis induction.

A standardized murine model of acute colitis induced by intrarectal administration of TNBS (95 mg/kg) was performed as previously described [51,55]. A group of mice was treated with the solvent alone (50% ethanol, control healthy mice). Mice were weighed prior and 48 h after colitis induction. Blood samples were obtained by retro-orbital bleeding and sera were stored at −20 °C until IL-6 measurement by ELISA (Duoset ELISA kits, R&D, Minneapolis, MN, USA). Colons were removed, washed and carefully opened for macroscopic inflammation grading performed blindly using the Wallace scoring method [56], reflecting both the intensity and the extent of the inflammatory lesions. Colonic sections (5-μm) fixed in 4% formaldehyde and embedded in paraffin were stained with hematoxylin and eosin (H&E) and histological scores were blindly recorded according to the Ameho criteria [57]. Colonic fragments were immediately stored in RNAlater^®^ buffer (Ambion, Life Technologies, Foster City, CA, USA) at −80 °C until gene expression analysis.

### 2.6. Quantification of Fecal Lipocalin 2 (Lcn-2)

Fecal samples were collected 48 h after colitis induction and homogenized using Lysing Matrix D (MPbio, Eschwege, Germany) in PBS containing 0.1% Tween 20 (100 mg/mL). The samples were centrifuged for 10 min at 12,000× *g* rpm at 4 °C and clear supernatants were collected and stored at −20 °C until analysis, as reported by Chassaing et al. [58]. Supernatants were diluted (50-fold to 10,000-fold, depending on severity of the colitis) and Lcn-2 levels were measured by ELISA using the Duoset kit (R&D System, Minneapolis, MN, USA), according to the manufacturer’s instructions.

### 2.7. Real-Time Quantitative PCR (qRT-PCR)

After homogenization of colonic samples, using Lysing Matrix D (MPbio, Eschwege, Germany), total RNA was extracted using Macherey-Nagel NucleoSpin RNAII isolation kit (Düren, Germany) according to the manufacturer’s recommendation. RNA quantity and quality were checked by Nanodrop (260/280 nm, 260/230 nm), and complementary DNA were prepared by reverse transcription of 1 μg total RNA using the High Capacity cDNA Reverse Transcription Kit (Applied Biosystems, Woolston Warrington, UK). Amplifications were performed using the Power SYBR Green PCR Master Mix (Applied Biosystems) on the QuantStudio™ 12K Flex Real-Time PCR System (Applied Biosystems, NJ, USA). Relative gene expressions (2^−∆∆ct^) were determined by comparing the PCR cycle thresholds (Ct) for the gene of interest and for the house keeping gene TATA-box-binding protein (*Tbp*; ΔCT), as described previously [38]. Primers sequences used in the study are presented in Table S1.

### 2.8. Immunofluorescence Staining and Confocal Analysis

Paraffin embedded sections of colon samples were deparaffinized using standard protocol. After antigen retrieval (Citrate buffer 10 mM pH 6 for 30 min at 100 °C), permeabilization in PBS/0.01% Triton, slides were blocked with PBS-SVF 5% and incubated overnight at 4 °C with anti-mouse Zona Occludens 1 (ZO-1), Claudin-2 or Claudin-3 primary rabbit antibodies at 1/50 dilution (Invitrogen, Rockford, IL, USA). After appropriate washings, goat anti-rabbit secondary antibody conjugated with Alexa Fluor 488 (Invitrogen) was used at 1/200 dilution for 2 h at room temperature (RT). The stained sections were subsequently labeled with DAPI (Invitrogen, 10 μg/mL) for 20 min at RT and slides were mounted using Dako fluorescent mounting medium. Imaging was performed using an inverted line-scanning microscope system equipped with a GaAsP detector and oil-immersion objectives (EC Plan Neofluar 40× oil/1.30; LSM880; Zeiss).

### 2.9. DNA Extraction from Stool and P. distasonis Quantification by qPCR

Colonic stool samples from individual mouse were collected, immediately frozen in liquid nitrogen and subsequently stored at −80 °C. DNA (100–200 mg/mouse) was isolated using Qiagen QIAamp DNA Stool Mini Kit. DNA quantity and quality were checked by Nanodrop (260/280 nm, 260/230 nm). The abundance of *P. distasonis* bacteria in stools was measured by qPCR using the Power SYBR Green PCR Master Mix (Applied Biosystems) on the QuantStudio™ 12K Flex Real-Time PCR System (Applied Biosystems) using 20 ng DNA and the following specific forward and reverse primers: *P. distasonis* (TGATCCCTTGTGCTGCT and ATCCCCCTCATTCGGA) and universal primers for all Eubacteria (ACTCCTACGGGAGGCAGCAGT and ATTACCGCGGCTGCTGGC) [59]. Relative amounts of DNA for *P. distasonis* for each mouse were determined using comparative cycle threshold method with Eubacteria as a control.

### 2.10. Preparation of Bacteria-Primed Bone Marrow Derived Dendritic Cells (BMDCs) and Co-Culture with Naive CD4^+^ T Cells

Bacteria-primed bone marrow derived dendritic cells (BMDCs) were differentiated from the bone marrow of BALB/c mice as described previously [60,61]. On day 9 after differentiation, cells were stimulated by bacteria (PF-BaE5 and PF-BaE11 strains) at a ratio bacteria/cells 10:1 for 5 h (gene expression analysis) or 24 h (flow cytometry analyses of co-stimulatory markers, co-culture with CD4^+^ T cells). Lipopolysaccharide (LPS *E. coli* serotype O111:B4; 1 μg/mL, Sigma Aldrich) was used as a positive control.

Naive CD4^+^ T cells were purified from spleens of BALB/c mice using the CD4 isolation kit (Miltenyi Biotec, Auburn, CA, USA). After 24 h stimulation with bacteria, BMDCs were harvested, washed and cocultured with naive CD4^+^ T cells at ratio DC/T cells: 1:10, in RPMI media supplemented by FBS 10%, glutamine 2 mM, gentamicin 50 μg/mL for 3 days (for RNA isolation and qPCR) and 7 days (for flow cytometry analysis). Dynabeads Mouse T-Activator CD3/CD28 (11452D, Life Technologies, Carlsbad, CA, USA), pan activator of T cells, were added (at a ratio beads/T cells of 1:1) to promote the polarization of bacteria-primed BMDCs on T cell subset.

RNA was extracted from BMDC (after 5 h stimulation) or CD4^+^ T cells (3 days co-culture) using RNeasy Mini Kit (Qiagen, Hilden, Germany) according to the manufacturer recommendation. Gene expression was quantified as described previously [62]. Briefly, total RNA was reverse transcribed using a High Capacity cDNA Reverse Transcription Kit (ThermoFisher Scientific, Waltham, MA, USA). Gene expression was quantified using TaqMan gene expression assays (ThermoFisher Scientific, Waltham, MA, USA) and the following primers and probes: *Beta Actin* as an endogenous control gene (Mm00607939_s1), *Il33* (Mm00505403_m1) and *Ebi3* (Mm00469294_m1). Results were expressed as 2^−∆∆ct^ values as described previously [61].

After 24 h of stimulation, BMDCs were harvested, washed with PBS and stained using the following monoclonal antibodies: anti-CD11c PE-Cy7 (clone N418; eBioscience, San Diego, CA, USA), CD80 FITC (clone 16-10A1; eBioscience,), CD86 PE-Cy5 (clone: GL1; eBioscience, San Diego, CAUSA), MHCII PE (clone M5/114.15.2; eBioscience, San Diego, CA, USA) and acquired using BD FACS Canto II (Becton Dickinson, Franklin Lakes, NJ, USA). On day 7 of the co-culture CD4^+^ T cells/bacteria primed BMDCs, GolgiPlug (BD Pharmingen, Franklin Lakes, NJ, USA) was added for 4 h to prevent extracellular secretion of cytokines. Cells were then washed with PBS and stained by the following antibodies: anti-CD4 FITC (clone GK1.5; BioLegend, San Diego, CA, USA), FoxP3 PE (clone NRRF-30; eBiosciences, San Diego, CA, USA). Intracellular staining of IL-10 (permeabilization and fixation) was performed using the Transcription Kit (Becton Dickinson, Franklin Lakes, NJ, USA) and anti-IL-10 (clone JES5-16E3; BioLegend, San Diego, CA, USA) according to the manufacturer recommendation. Data were analyzed using FlowJo (Becton Dickinson, Franklin Lakes, NJ, USA).

### 2.11. Statistical Analysis

Graph preparation and statistical evaluation were performed using GraphPad Prism software. Statistical significance was determined using non-parametric one-way analysis of variance (ANOVA) followed by Dunnett’s multiple comparison posthoc test and non-parametric two-way ANOVA with Dunnett’s post-tests (GraphPad Prism software, version 7.00). Data with *p* values ≤ 0.05 were considered to be significant.

## 3. Results

### 3.1. P. distasonis Strains Displayed Different Ability to Restore the H_2_O_2_-Induced Disruption of the Epithelial Barrier

**Figure 1:**
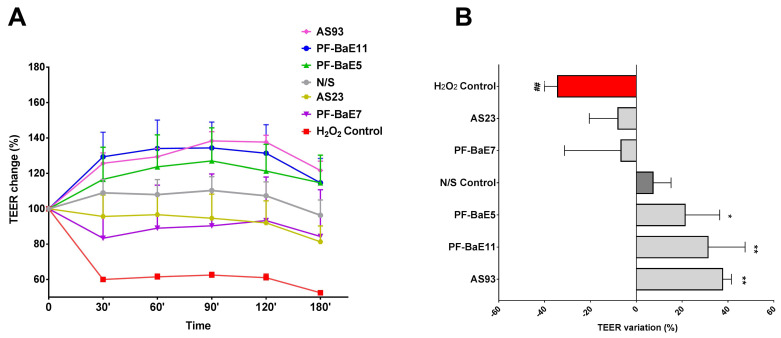
Ability of the strains to restore the H_2_O_2_-induced disruption of the epithelial barrier. The Caco-2 confluent monolayers were pre-treated (or not) with the bacteria at the apical side (10:1 bacteria/cell ratio) for 30 min and sensitized with H_2_O_2_ (100 μM). Trans-epithelial electrical resistance (TEER) was measured at T0 and every 30 min. Data represent (**A**) the time-dependent means of relative changes (in %) of TEER ± SEM in comparison to TEER at T0 and (**B**) the final % TEER variations at 120 min. ^#^ and * refer to the comparison of the cells treated with H_2_O_2_ (H_2_O_2_ Control) and without H_2_O_2_ (N/S Control) or bacteria-treated cells with H_2_O_2_ versus H_2_O_2_ Control; * *p* < 0.05; ** or ^##^ *p* < 0.01.

We evaluated the ability of the selected strains to restore or strengthen the gut barrier function, by using an in vitro epithelial barrier model as previously described [38,63]. Polarized Caco-2 cell monolayers were exposed (or not) to the bacteria and were sensitized (or not) with H_2_O_2_. As expected, treatment of epithelial monolayers with H_2_O_2_ induced an increased permeability, as determined by a decrease of the TEER in a time-dependent manner (Figure 1A), in comparison to untreated cells. *P. distasonis* strains PF-BaE7 and AS23 were able to partially restore the epithelial barrier, the TEER being higher than the level observed with H_2_O_2_, however not in a significant manner (Figure 1A,B). Interestingly, the three strains *P. distasonis* PF-BaE5, PF-BaE11 and AS93 were able to reinforce in a significant manner, the epithelial barrier, the TEER being higher than non-treated monolayers (Figure 1A,B).

### 3.2. P. distasonis PF-BaE5 and PF-BaE11 Exhibit the Best Anti-Inflammatory In Vitro Profile

**Figure 2:**
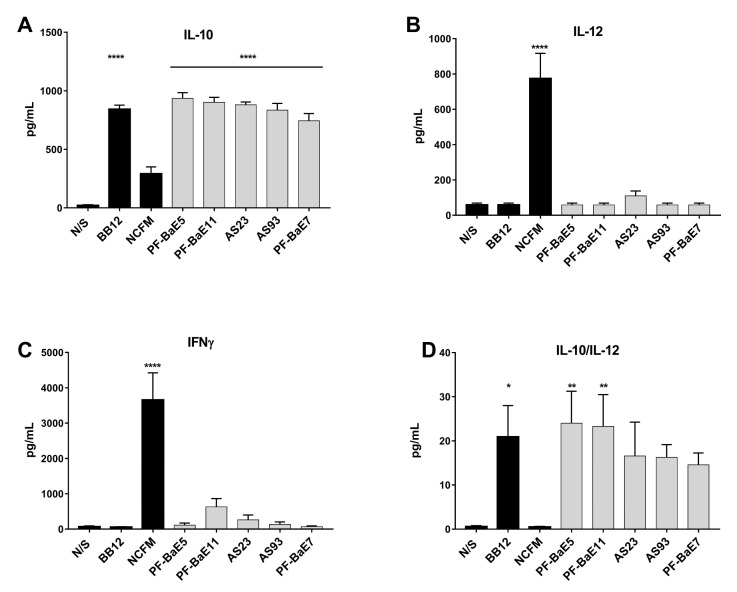
In vitro immunomodulatory profiles of the strains. (**A**) IL-10, (**B**) IL-12p70 and (**C**) IFN-γ production was evaluated in the supernatants of peripheral blood mononuclear cells (PBMCs; *n* = 5 different donors) stimulated for 24 h by the tested strains or two control strains (*L. acidophilus* NCFM and *B. animalis* subsp. *lactis* BB12), in comparison to non-treated cells (N/S). (**D**) IL-10/IL-12 ratio was calculated. Data represent means ± SEM of the 5 independent donors. * refers to the comparison of bacteria-stimulated PBMCs versus untreated cells; * *p* < 0.05, ** *p* < 0.01, **** *p* < 0.0001.

To investigate the immunomodulatory capacities of the strains, human immune cells (PBMCs) were stimulated in vitro by the different bacteria (ratio 10:1) and their ability to induce the release of the anti-inflammatory cytokine IL-10 (Figure 2A) or the pro-Th1 IL-12 (Figure 2B) and IFNγ (Figure 2C) cytokines was measured by ELISA. None of the strains induced a significant production of IL-12 (between 58 and 110 pg/mL) in comparison with untreated cells, while the pro-Th1 *L. acidophilus* NCFM reference strain induced significant levels (775 pg/mL). All the *P. distasonis* were also low inducers of IFNγ (between 66 and 630 pg/mL), at levels much lower than those obtained for the pro-Th1 *L. acidophilus* NCFM strain (3655 pg/mL) [51]. The 5 strains were very high inducers of IL-10 with similar or higher levels (between 744 and 936 pg/mL; *p* < 0.001) than the anti-inflammatory control strain *Bifidobacterium animalis* spp. *lactis* BB12 [52] (which induced 842 pg/mL IL-10). The calculation of the IL-10/IL-12 ratio (Figure 2D) highlighted PF-BaE5 and PF-BaE11 as the two strains exhibiting an elevated and significant anti-inflammatory in vitro profile, while PF-BaE7 (also isolated from newborn sample) displayed the lowest anti-inflammatory effect.

On the basis of these in vitro results, we observed that *P. distasonis* strains PF-BaE11 and PF-BaE5 exhibited the strongest anti-inflammatory profile together with a good ability to restore the epithelial barrier, while AS93 had the strongest effect on the epithelial barrier model and strongly stimulated the IL-10 production even if the IL-10/IL-12 ratio was not significant compared to untreated cells.

### 3.3. Strains Survive Differently to Gastric Condition

**Figure 3:**
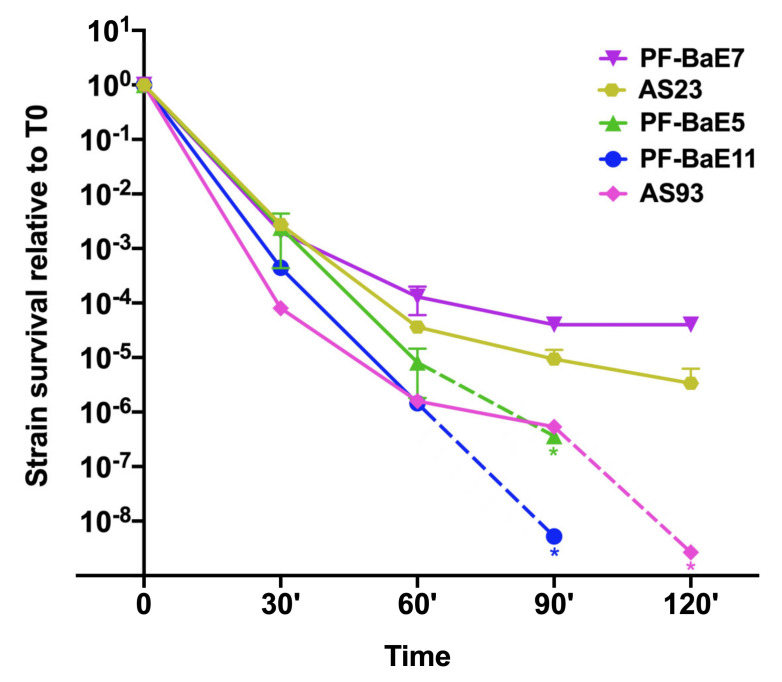
Relative survival of selected strains to the simulated gastric fluid over 2 h. Results are expressed as the ratio of the CFU/mL at a given time point to the CFU/mL at time zero ± SEM with a semi-logarithmic scale. * indicates a default value corresponding to a number of CFU/mL below 100 (as the CFU/mL at T0 was slightly different among strains, the default values differ). Dotted lines are joining the last CFU measures and default values.

The capacity of bacteria to survive to gastric conditions is an important technical criterion in the selection of probiotic strains. Prior to in vivo evaluation of the strains ability to alleviate colitis, we compared their tolerance to a gastric stress. The bacterial suspensions were incubated in the simulated gastric fluid (SGF) for 2 h with plating at different times of incubation. None of the strains fully survived to the gastric condition and marked differences were observed among strains (Figure 3). We could roughly distinguish two groups of strains. (i) PF-BaE7 and AS23 were the most SGF tolerant with a 5 and 6 log decreases in viability at 120 min exposure and (ii) PF-BaE5, PF-BaE11 and AS93 were more sensitive to SGF exposure with less than 10^2^ CFU/mL after 90 min (PF-BaE5 and PF-BaE11) or 120 min (AS93) exposure to SGF. Of note, the difference in the survival of the different strains increased with the duration of the stress. After 30 min exposure to SGF, the survival difference between the most tolerant (PF-BaE5) and the most sensitive strain (AS93) was only 30 fold but at 90 min and 120 min, this difference reach 6500 fold between the most tolerant strain (PF-BaE7) and the most sensitive one (PF-BaE11 less than 100 CFU/mL at 90 min). Considering the heterogeneity of the strains survival during the gastric stress, we added sodium bicarbonate to the bacterial pellets before gavage to mice to neutralize the gastric pH and avoid a bias possibly hampering the comparison of strains effects in the in vivo assay.

### 3.4. P. distasonis PF-BaE5, PF-BaE11 and AS93 Were the Most Potent Strains to Counteract Inflammation in a Murine Model of TNBS-Induced Colitis

**Figure 4:**
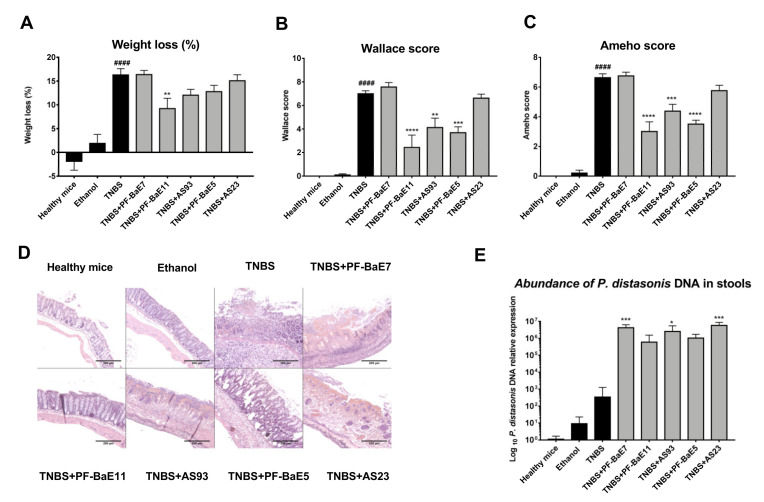
Ability of *Parabacteroides distasonis* strains to counteract the acute TNBS-induced colitis. (**A**) Body weight loss (as a percentage of the initial weight). (**B**) Macroscopic evaluation of colonic inflammation (Wallace score). Percentages of protection are indicated above each bar. (**C**) Histologic evaluation of colonic inflammation (Ameho score). (**D**) Representative histological sections (stained by H&E, 100× magnification) of mice treated with TNBS (TNBS) or not (healthy mice, ethanol-control mice) and orally treated with the selected strains. Data represent means of each group (*n* = 9 mice per group) ± SEM. ^#^ and * refer to the comparisons of TNBS versus healthy mice or bacteria-treated group versus TNBS control group, respectively; ** *p* < 0.01, *** *p* < 0.001, ^####^ or **** *p* < 0.0001. (**E**) Abundance of *P. distasonis* specific DNA in the stools collected two days after colitis induction and evaluated by qPCR. Results are expressed as relative expression compared with values obtained from healthy mice. Data represent means of each group (*n* = 9 mice per group) ± SEM. * refer to the comparisons of each group versus healthy mice; * *p* < 0.05, *** *p* < 0.001.

**Figure 5:**
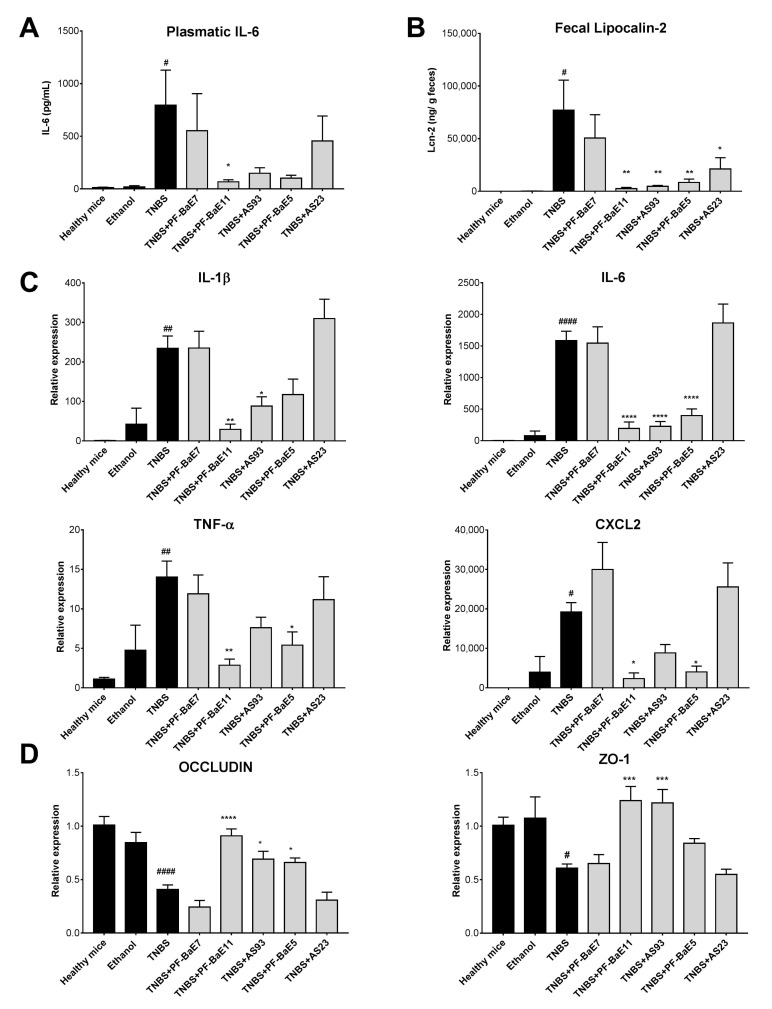
Capacity of the strains to modulate (**A**) the plasmatic IL-6 concentration (pg/mL), (**B**) the fecal lipocalin-2 levels (μg/g feces), (**C**) the expression of genes encoding proinflammatory markers or (**D**) tight junction proteins during TNBS-induced colitis. IL-6 and fecal lipocalin-2 concentrations were measured by ELISA. Gene expression of *Il1b, Il6, Tnfa*, *Cxcl2, Occludin* and *Zo1* was evaluated by qRT-PCR from colonic samples obtained two days after colitis induction. Results are expressed as Relative expression compared with values obtained from healthy mice. Data represent means of each group (*n* = 9 mice per group) ± SEM. ^#^ and * refer to the comparisons of the TNBS-treated control versus healthy mice or bacteria-treated group versus TNBS control group, respectively; ^#^ or * *p* < 0.05, ^##^ or ** *p* < 0.01, *** *p* < 0.001, ^####^ or **** *p* < 0.0001.

We evaluated the in vivo protective efficacy of the five selected *P. distasonis* strains using a well-established murine model of acute colitis [55] induced by a single rectal administration of TNBS (95 mg/Kg). As expected, TNBS administration induced a strong colitis resulting in significant body weight loss (by 16.3 ± 1.33%, Figure 4A), high macroscopic (Wallace score of 7 ± 0.25; *p* < 0.0001; Figure 4B) and histological (Ameho score of 6.6 ± 0.2; Figure 4C,D) scores of inflammation, correlated with a significant increase in the plasmatic level of IL-6 (795 pg/mL; *p* < 0.05; Figure 5A) and of the expression of all the proinflammatory genes tested (Figure 5C; *p* < 0.05 to 0.0001). Three strains were able to alleviate acute colitis. *P. distasonis* PF-BaE11 was the most protective strain, as shown by the lowest weight loss (9.22 ± 2.14; *p* < 0.01), reduction of the macroscopic (2.43; *p* < 0.0001) and histological scores (3 ± 0.65; *p* < 0.0001) of inflammation, leading to a protection of 65% (Figure 4A–D). The anti-inflammatory properties of the PF-BaE11 strain was confirmed by a significant decrease of the plasmatic IL-6 level (67 ± 19.5 pg/mL; *p* < 0.05) in comparison to TNBS control mice (Figure 5A) and its ability to significantly limit the expression of the proinflammatory genes, Il1b (*p* < 0.01), Il6 (*p* < 0.0001), Tnfa (*p* < 0.01) and Cxcl2 (*p* < 0.05; Figure 5C). *P*. *distasonis* PF-BaE5 also significantly protected mice from colitis, as shown by the significant decrease in the macroscopic (3.68 ± 0.5; *p* < 0.0001) and histological (3.5 ± 0.26; *p* < 0.0001) score of inflammation and leading to a 47% protection. This was correlated by a decreased IL-6 level, although not in a significant manner (Figure 5A) and a significant downregulation of the expression of Il6, Cxcl2 and Tnfa genes and to a lesser extend Il1b (Figure 5C). *P. distasonis* AS93 was also able to alleviate inflammation, conferring a protective effect of 41% with a significant decrease in the Wallace (*p* < 0.01), Ameho (*p* < 0.001) scores of inflammation and a significant downregulation of Il1b (*p* < 0.05) and Il6 (*p* < 0.0001) genes expression. In the opposite *P. distasonis* PF-BaE7 and AS23 displayed no protective effect.

We confirmed by qPCR that the levels of *P. distasonis* bacterial DNA in the stools increased in all groups of mice, which received the different strains, in comparison to control healthy mice or mice treated with TNBS only (Figure 5). Interestingly, the protection level was not linked to the increased level of the bacteria. The three most protective strains (*P. distasonis* PF-BaE11, PF-BaE11and AS93) were detected by qPCR at a lower level than the two other strains (PF-BaE7 and AS23), which did not alleviate the colitis.

The results were confirmed with the fecal level of lipocalin 2 (Figure 5B), which was significantly elevated in TNBS control mice (*p* < 0.05) in comparison to healthy control mice, while significantly decreased in mice treated with the three protective strains, *P. distasonis* PF-BaE11 (*p* < 0.001), PF-BaE5 and AS93 (*p* < 0.05). As expected, no significant decrease in this proinflammatory marker was observed in PF-BaE7 and AS23-treated mice. Interestingly, while TNBS treatment induced a significant drop in the expression of the tight junction protein encoding genes Occludin and Zo1 in comparison to healthy mice (Figure 5D; *p* < 0.0001 and 0.05, respectively), *P. distasonis* PF-BaE11 and AS93 were able to significantly restore their expression (*p* < 0.05–0.001) while PF-BaE5 only restore Occludin expression in a significant manner (*p* < 0.05), confirming the ability of the strains to strengthen the gut barrier, as highlighted in the in vitro epithelial barrier model. In contrast, strains PF-BaE7 and AS23 exhibited no ability to improve the epithelial barrier function in vivo.

**Figure 6:**
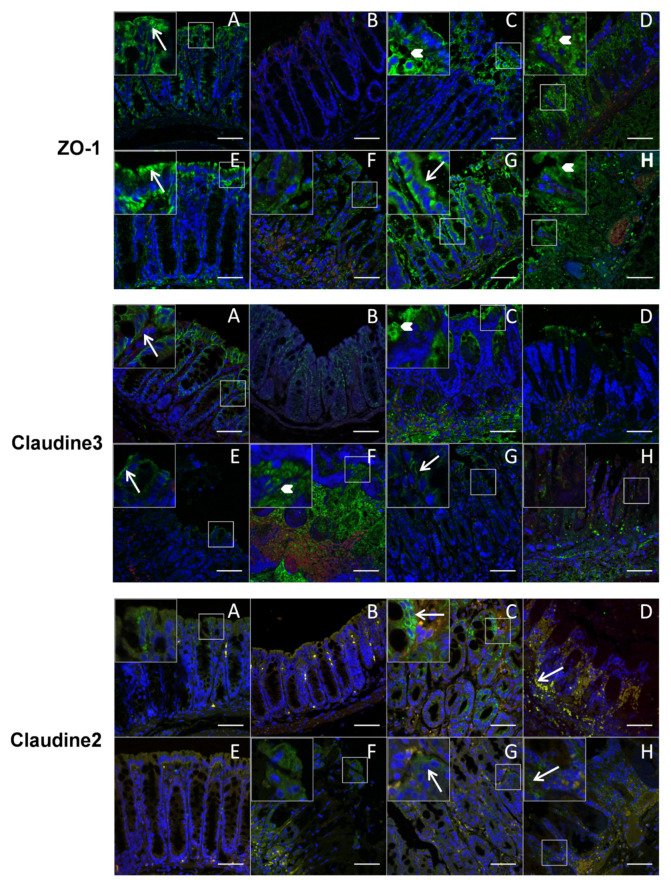
Ability of the strains to modulate tight junction proteins in the colon. Representative immunofluorescent images of ZO-1, Claudin-3 and Claudin-2 of colon sections of the different groups of mice (**A**): healthy mice; (**B**): ethanol, (**C**): TNBS; (**D**): TNBS+PF-BaE7; (**E**): TNBS+PF-BaE11; (**F**): TNBS+AS93; (**G**): TNBS+PF-BaE5 and (**H**): TNBS+AS23, observed by immunofluorescence and confocal microscopy after labeling with specific primary antibodies and AF488-conjugated secondary antibody (green), with nuclei counterstained with DAPI (blue) stain. A red canal was added to identify autofluorescence tissue elements (red/yellow) and reinforce the specific labeling. Boxed areas represent magnified images (×2.5) as insets from the corresponding white box. Long arrows indicate normal TJ (ZO-1 and Claudin-3) distribution at the apical and lateral levels and labeling for Claudin-2; short arrows indicate discontinuities or diffuse cytoplasmic distribution of TJ. Scale bars 50 μm.

The results were corroborated by immunofluorescence labeling of tight junction proteins (Figure 6). Even if the fluorescence intensity of ZO-1 and Claudin-3 was not clearly different among the different groups of mice, they were more expressed in luminal and apicolateral membrane localization (see long arrows and magnified areas) in control healthy mice and protected mice treated with PF-BaE11, PF-BaE5 and AS93 strains) while a loss in lateral membrane locations appeared in TNBS-treated mice and in non-protected animals (treated with PF-BaE7 and AS23 strains), with a more diffuse cytoplasmic localization and discontinuities (see short arrows and magnified areas) as observed in patients suffering from CD [64,65]. In contrast, Claudin-2 immunoreactivity, which is recognized as a marker of leaky epithelia [64], was lower and predominantly luminal in the healthy mice control (treated or not with ethanol) and protected mice treated with *P. distasonis* PF-BaE11 and AS93, while it was increased in TNBS control mice and non-protected mice (PF-BaE7 and AS23) and highly labeled at the bottom of the crypt and basolateral membrane, indicating a leaky gut associated with modulation of TJ distribution.

### 3.5. P. distasonis PF-BaE5 and PF-BaE11 Led to Immature Bone Marrow Dendritic Cells (BMDCs) In Vitro and Regulatory T Lymphocytes When Co-Cultured with Naïve CD4^+^ T Cells

**Figure 7:**
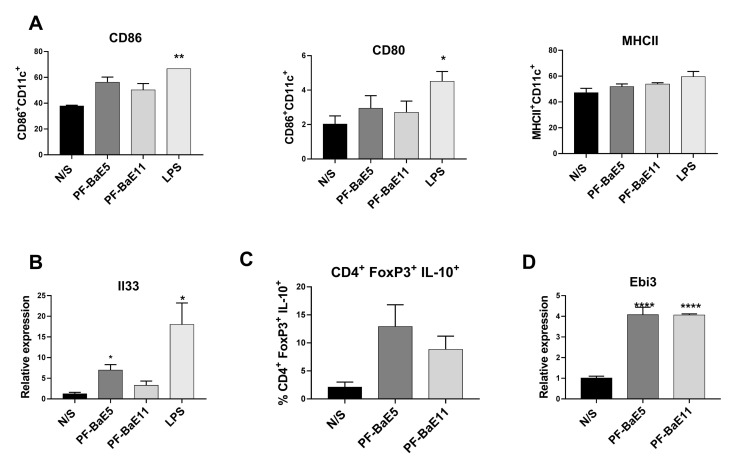
Ability of the strains (**A**) to activate murine BMDCs and to induce (**B**) the expression of Il33 in BMDC, (**C**) the induction of IL-10 producing CD4^+^ FoxP3^+^ T cells and (**D**) the gene expression of Ebi3 in CD4^+^ T cells. Data represent means ± SEM of 3 independent experiments. * refers to the comparison of bacteria-treated cells versus unstimulated cells (N/S); * *p* < 0.05, ** *p* < 0.01, **** *p* < 0.0001.

To get better insight into the mechanisms involved in the anti-inflammatory abilities of the two most potent *P. distasonis* strains PF-BaE5 and PF-BaE11, we investigated the ability of the two strains to activate murine BMDCs in vitro. The two strains induced very low expression levels of co-stimulatory markers (non significant) as compared with untreated DCs (N/S), while Lipopolysaccharide (LPS) induced moderate but significant levels of CD86 and CD80 (Figure 7A). Both strains were able to upregulate Il33 gene expression (Figure 7B), which was significant only for PF-BaE5 (*p* < 0.05), while proinflammatory genes remained unmodified (data not shown). This led us to further evaluate the immune-regulatory capacity of the strains by following their impact on the polarization of CD4^+^ T cells. To this end, bacteria-primed BMDCs were co-cultured with naïve CD4^+^ CD25^−^ T cells for 7 days and the phenotype of polarized T cells was analyzed by flow cytometry. While no polarization towards Th1 or Th2 was observed (data not shown), BMDCs primed with both strains were able to promote the differentiation of regulatory T cells, highlighted by an increased level of IL-10 producing CD4^+^ FoxP3^+^ (Figure 7C). Interestingly, we observed a strong and significant increased gene expression of the Epstein Barr virus-induced gene 3 (Ebi3) in the CD4^+^ T cells obtained after co-culture with bacteria-primed DC (Figure 7D). This led us to suggest that the two *P. distasonis* strains could induce immature regulatory DCs able to promote the induction of regulatory T cells.

## 4. Discussion

The last decades of research on the microbiome and IBD started with descriptive works highlighting that dysbiosis plays a substantial role in the pathophysiology of the disease, and are now opening the way for innovation through bacteria-specific targeted therapies to prevent and treat inflammatory diseases [66]. Such strategies are now under intensive study and NGPs or LBPs start to be developed as new therapeutic tools. For instance, the use of *F. prausnitzii* [49], a bacteria depleted in the microbiome of Crohn’s patients compared with those of healthy individuals appears as a promising strategy. However, the extreme oxygen-sensitivity of *F. prausnitzii* and other intestinal microbiota members making notoriously difficult to cultivate and to preserve them, remains a challenge for the development of LBP formulations. Moreover, these LBPs commensal candidates must meet the same criteria than the “traditional” probiotics: They have to be well-characterized, with clear health beneficial effects on the host, their safety must be established and technological criteria such as biomass production and tolerance to long term storage should be met. Currently despite very active research in this field, identifying functional strains remains a critical step to better understand the host microbiota interactions and to select promising bacteria to be used as LBPs. IBD is associated with a leaky gut and increased permeability favoring the maintenance of chronic inflammation and immune imbalance [67]. Health promoting bacteria able to exhibit anti-inflammatory capacity as well as the ability to restore the gut barrier function would therefore represent interesting LBPs against IBD.

*P. distasonis* strains belongs to the human core intestinal microbiota as shown (i) using shotgun metagenomics for the identification of the 57 species shared by 90% of the intestinal microbiome of 184 individuals originated from China, Denmark and France [2] and (ii) through the 16-S sequencing identification of the 17 genera shared by 95% of 2241 samples from the Western Europe population [27]. These core genera and species are suggested to have important physiological function for the host. Of interest, *P. distasonis* has been reported in different studies to be more frequently absent in patients with IBD than in control subjects and to be significantly decreased in inflamed tissue compared to uninflamed sites [68,69]. In addition, a combinatorial approach based on the transfer of different bacterial consortia generated from human fecal microbiota to recipient germ-free mice enable to select gut microbial communities able to influence specific physiologic phenotypes [66]. This study notably allowed us to identify strains promoting immune regulatory responses or influencing gut metabolic phenotypes after monocolonization, in particular different *Bacteroides* and one *P. distasonis* strain. Therefore, in the present work, we investigated the health-promoting potential of five strains of *P. distasonis* isolated from human feces using a combination of in vitro and in vivo models. We previously used these in vitro models to select “traditional” putative probiotic strains according to their ability to improve the gut barrier and to exhibit anti-inflammatory profile, showing the rational to combine such in vitro test to select potent strains able to alleviate colitis in mice [38,39]. We highlighted the potential of three different strains, *P. distasonis* PF-BaE11, PF-BaE5 and AS93, which were able to combine the best abilities in vitro: a strong capacity to strengthen the epithelial barrier and to induce the anti-inflammatory cytokine IL-10. Even if some limited studies reported a potential role of IL-10 for the pathogenesis of human inflammation [70,71], IL-10 is well known for its ability to weaken Th1 and Th17 proinflammatory responses, notably by reducing DC-derived IL-12 and IL-23 production [72]. Therefore, we unraveled in vivo the protective abilities of the strains in a TNBS-induced murine model of acute colitis. Previously standardized for traditional probiotic strains [55]. Since we observed that sensitivity to the gastric conditions strongly differed upon strains (Table 2), it prompted us to test them in conditions neutralizing the gastric pH to ensure a better comparability.

Interestingly, the two strains PF-BaE5 and PF-BaE11, which exhibited the best in vitro anti-inflammatory profile together with a strong ability to restore the epithelial barrier, were the most potent strains in alleviating intestinal inflammation, these strains being able to significantly rescue mice from colitis. *P. distasonis* AS93, which also displayed a strong ability to restore the gut barrier and significant stimulation of IL-10, was also able to confer a protective effect, however to a lesser extent than the two other protective strains (Table 2). The strains AS23 and PF-BaE7, which were less potent in their in vitro capacity to strengthen the epithelial barrier and to induce the release of IL-10, were not protective in this model. Interestingly, as previously reported, intestinal inflammation induced by TNBS led to an increase of the pore-forming Claudin-2, a structural component of tight junction complex recognized as a mediator of “leaky gut”, increasing the passage of macromolecules through the epithelial barrier [64]. Claudin-2 has been shown to be highly detected in the bottom of intestinal crypts in samples from patients with active CD [72], as we observed in TNBS mice. The level of Claudin-2 was lower in protected mice treated with PF-BaE5, PF-BaE11 and AS99 while it was similar as control TNBS mice in animals treated with AS23 and PF-BaE7 strains. This work allowed us to determine that selected *P. distasonis* species exhibited promising potential in alleviating intestinal inflammation, but in a strain-dependent manner and also provide crucial clues for their selection, even if in vitro results do not totally predict their in vivo protective behaviors.

Beneficial impact of probiotics and/or LBPs can be dependent on or can be predicted by the host microbiota composition, which can resist differently to the colonization of exogenous bacterial strains [73], suggesting that it is not only important to better understand the host–microbiota interaction but also take into account the microbiota composition for the design of individualized nutritional intervention [74].

With the purpose of understanding the mode of action, we showed that the two most efficient strains PF-BaE5 and PF-BaE11 promoted regulatory DCs and Tregs in vitro. This was associated to an upregulation in BMDCs of the gene expression of *Il-33*, a member of the IL-1 family, known to have a key role in innate and adaptive immunity, in balancing Th2 and Th1 cells versus Tregs [75]. Indeed, we were able to show that PF-BaE5 and PF-BaE7-primed DCs promoted the polarization of CD4^+^ T cells towards a regulatory phenotype (CD4^+^ FoxP3^+^ IL-10^+^) with an increased expression of the gene encoding Ebi3. Ebi3 is a subunit of the heterodimeric cytokine IL-35, a potent immunosuppressive cytokine secreted by Tregs and B regulatory cells with a strong ability to inhibit T cell differentiation and effector functions [76]. IL-35-induced Treg cells (iTr35) produce more IL-35 [77] and type 1 Treg cells (Tr1) use IL-35 to suppress the immune response [78].

The oral administration of crude lysates of a pool of anaerobic bacteria isolated from mouse intestinal microbiota was able to significantly suppress Dextran Sodium sulfate (DSS)-induced colitis [79]. From the lysates tested, only the crude lysate obtained from a *P. distasonis* strain, and particularly its membranous fraction, was able to significantly alleviate the acute DSS colitis [80]. The protective effect was associated with an increased level in both CD4^+^ FoxP3^+^ and CD4^+^ FoxP3^−^ T regulatory cells in mesenteric lymph nodes of treated mice. Our work confirms that selected *P. distasonis* strains are able to induce regulatory DCs and Treg in vitro. It remains important to better decipher the mechanism of action, notably to further investigate the regulatory responses induced in vivo but also the signaling pathways involved, notably the potential role of IL-33 and Ebi3.

*P. distasonis* has also been reported to be a beneficial commensal gut microorganism in different pathophysiology models through anti-inflammatory and barrier restoration abilities. A significant depletion of this species has been observed in tumor-bearing mice in an experimental model of colon cancer, obesity-driven in genetically deficient mice (Apc1638N) and its abundance was inversely correlated with colonic interleukin IL-1β levels [81]. These results were recently confirmed in another model in mice receiving the carcinogen azomethane in which increased colonic expression of IL-10 and TGF-β together with an increased levels of tight junction proteins ZO-1 and occludin at the transcript and protein levels [82]. *P. distasonis* appeared also significantly reduced in multiple sclerosis (MS) in human patients and exposing PBMCs from healthy donor to *P. distasonis* extracts significantly increased the percentage of CD25^+^ IL-10^+^ T lymphocytes, including IL-10^+^ Tr1 regulatory (Treg) cells. Colonization of antibiotic-treated or germ-free mice with a single *P. distasonis* strain induced Tregs differentiation [83]. This confirmed previous study performed in gnotobiotic mice colonized with *P. distasonis* showing an induction of Treg differentiation [84]. A strain of *P. distasonis* was shown to reduce neuroinflammation in vitro by exhibiting a strong capacity to reduce IL-6 secretion, but also antioxidant capacity on different brain cell lines [85]. All these results highlight the potential anti-inflammatory effect of this bacterium at multiple levels and its ability to promote intestinal barrier integrity, as we observed in our models.

However, some reports suggested that *P. distasonis* strains could contribute to the development of chronic diseases. A particular strain (CavFT-hAR46) has been isolated from a gut intramural cavernous fistulous tract microlesion from the gut wall of a CD patient. The authors suggested it to be a potential pathogenic strain, however they did not demonstrate its capacity to promote intestinal inflammation [86]. Peptidoglycan recognition proteins (*Pglyrps*) have been shown to participate in maintaining intestinal microbiota and mice deficient in the encoding genes were more sensitive to colitis and presented an increased level of *P. distasonis* and *Prevotella falsenii* [59]. Treatment of wild type mice depleted of their intestinal microbiota by a 3-weeks antibiotic treatment with a strain of each species (*P. distasonis* ATCC8503 and *P. falsenii* 15124), developed more severe DSS-induced colitis, suggesting these strains to be colitis-promoting species. As mentioned by the authors, the exact knowledge of colitis-promoting and colitis-protective bacteria remains important in designing microbiota-based management of IBD. Indeed, our results highlighted strain-dependent protective abilities of *P. distasonis* to alleviate colitis, however we did not observe any exacerbating impact. Genomic comparison of strains exhibiting an opposite profile would greatly help understanding the mechanisms involved in respective health beneficial or detrimental abilities of commensal bacteria. Since membranous fraction of *P. distasonis* have been shown to display strong anti-inflammatory abilities [80], it should also be interesting to decipher the role of cell wall components, notably by comparing the structure of peptidoglycan of strains exhibiting opposite functional abilities, as we previously performed for lactobacilli, for which we identified the crucial role of specific muropeptides acting through a nucleotide binding oligomerization domain-containing protein 2 (NOD2) -dependent signaling [37].

Interestingly, a recent study on the infant gut microbiota determined that *Parabacteroides* is one of the most discriminative genera of full-term delivery and in particular of vaginally delivered newborns [87]. In this study, we characterized strains isolated from adults (AS23 and AS93) and newborns fecal samples (PF-BaE5, PF-BaE7 and PF-BaE11). *P. distasonis* PF-BaE5 and PF-BaE11 displayed the best anti-inflammatory effects in the colitis model. Although their colonization ability and full innocuity remained to be confirmed, our observations open the door to use these bacteria for live therapeutic perinatal interventions notably for preterm infants.

## 5. Conclusions

As one of the core gut commensal bacteria, the abundance of *P. distasonis* has been negatively correlated with many inflammatory chronic diseases including IBD. Moreover, the successful applications of *P. distasonis* in different experimental models of chronic diseases inspire therapeutic concepts of utilizing such an intestinal commensal strain to improve gut microbiota dysbiosis-associated diseases, even if some reports remain controversial. Our objective was to compare the health-promoting properties of five different *P. distasonis* strains. We showed that they can be highly effective bacteria in alleviating intestinal inflammation, however in a strain-dependent manner.

By combining in vitro and in vivo models, we highlighted the health beneficial abilities of three strains, which could be interesting as complementary therapies to maintain remission and improve the quality of life of patients suffering from IBD. Our work pointed out some suggested mechanisms, which remain important to better unravel. Notably it would be very important to determine which bacterial structure(s) could explain the differential abilities of the strains, but also better decipher the host immune responses and the impact of the LBPs on the gut microbiota composition. This could be achieved by comparing strains with different beneficial properties that would allow complete knowledge of this species of interest. Finally, it remains necessary to investigate the clinical efficacy and the safety of the most promising strains, which are mandatory to make sure no adverse effects would occur before final applications in human medicine.

## Figures and Tables

**Table 1:** Bacterial strains, growth media and origins.

Strains Designation	Species	Growth Medium	Origin
AS93	*Parabacteroides distasonis*	BHIS	Healthy adult feces
AS23	*Parabacteroides distasonis*	BHIS	Healthy adult feces
PF-BaE5	*Parabacteroides distasonis*	BHIS	Newborn fecal samples
PF-BaE7	*Parabacteroides distasonis*	BHIS	Newborn fecal samples
PF-BaE11	*Parabacteroides distasonis*	BHIS	Newborn fecal samples

**Table 2:** Summary of statistically significant effects of the *P. distasonis* strains in in vitro models and in vivo colitis model.

Strains	Improvement of Epithelial Barrier	IL-10 Induction	Anti-Inflammatory Profile (Il-10/IL-12)	Tolerance to Gastric Conditions (120 min)	In Vivo Protective Capacity
*P. distasonis* AS23	±	+++	+	++	−
*P. distasonis* AS93	+++	+++	+	±	++
*P. distasonis* PF-BaE5	++	+++	++	±	+++
*P. distasonis* PF-BaE7	±	+++	+	++	−
*P. distasonis* PF-BaE11	+++	+++	++	±	+++

## Supplementary Materials

The following are available online at https://www.mdpi.com/2073-4409/9/9/2104/s1, Table S1: Forward and reverse primers used in the study for the respective genes encoding β-actin, IL-1β, IL-6, TNF-α, CXCL-2, ZO-1 and Occludin.
