# Supplementary material for: In Vitro Characterization of Gut Microbiota-Derived Commensal Strains: Selection of Parabacteroides distasonis Strains Alleviating TNBS-Induced Colitis in Mice
Source: Cells. 2020 Sep 16;9(9):2104. doi: 10.3390/cells9092104 (PMC7565435; doi:10.3390/cells9092104)
Supplement: Supplementary file 1 [file cells-09-02104-s001.pdf]

Supplementary Table 1: Forward and reverse primers used in the study for the respective genes encoding TATA-Box Binding Protein, IL-1β, IL-6, TNF-α, CXCL-2, ZO-1 and Occludin.

Tbp-F	5′-TGGTGTGCACAGGAGCCAAG-3′
Tbp-R	5′-TTCACATCACAGCTCCCCAC-3′
Il1b-F	5′-TTGACGGACCCCAAAAGATG-3′
Il1b-R	5′-AGAAGGTGCTCATGTCCTCA-3′
il6-F	5′-AGCCAGAGTCCTTCAGAGAGATAC-3′
il6-R	5′- ATTGGATGGTCTTGGTCCTTAGC-3′
tnfa-F	5′-CCCTCACACTCAGATCATCTTCTC-3′
tnfa-R	5′-GGCTACAGGCTTGTCACTCG-3′
cxcl2-f	5′-CAAAAGATACTGAACAAAGGCAA-3′
cxcl2-R	5′-TCAGGTACGATCCAGGCTTCC-3′
zo1-F	5′-GACTCCAGACAACATCCCGAA-3′
zo1-R	5′- ACGCTGGAAATAACCTCGTTC 3′
occludin-F	5′-TCAGGGAATATCCACCTATCACTTCAG-3′
occludin-R	5′-CATCAGCAGCAGCCATGTACTCTTCAC-3′
